# The origin of p40-negative and CDX2-positive primary squamous cell carcinoma of the stomach: case report

**DOI:** 10.1186/s12957-019-1594-8

**Published:** 2019-03-19

**Authors:** Yukinori Yamagata, Kazuyuki Saito, Shinichi Ban, Akiko Fujii, Masatoshi Oya

**Affiliations:** 1 Department of Surgery, Dokkyo Medical University Saitama Medical Center, Koshigaya, Japan; 2 Department of Gastric Surgery, National Cancer Center Hospital, 5-1-1 Tsukiji, Cyuo-ku, Tokyo, 104-0045 Japan; 3 Department of Pathology, Dokkyo Medical University Saitama Medical Center, Koshigaya, Japan

**Keywords:** Case report, Gastric cancer, Squamous cell carcinoma, Cytokeratin 5/6, p63, p40, CDX2

## Abstract

**Background:**

Primary gastric squamous cell carcinoma (SCC) is a very rare disease. The origin of this tumor remains unclear, although there are some hypotheses.

**Case summary:**

A 60-year-old man consulted a previous physician complaining of upper abdominal pain. Esophagogastroduodenoscopy revealed type 2 gastric cancer, and the patient was referred to our hospital. After close examination, the patient was diagnosed as cStage IIA gastric adenocarcinoma, and distal gastrectomy was performed. Histochemical studies showed typical findings of SCC, and the tumor was surrounded by intestinal metaplasia. Immunohistochemical examination was positive for cytokeratin (CK) 5/6 and caudal-type homeobox protein 2 (CDX2) and negative for p63/p40.

**Conclusion:**

The results of immunostaining for CK5/6 supported that this tumor was SCC, but the question why p63/p40 were negative and CDX2 was positive still remained. Concerning about the origin of p63/p40 and CDX2, it was suggested that the tumor cells were not derived from ectopic squamous epithelium but from intestinal metaplasia. And tumor cells looked like homogeneous and squamous metaplasia was not observed. These findings supported the idea that these tumor cells arose from stem cells in the intestinal metaplasia of the stomach.

## Introduction

According to the Japanese classification of gastric carcinoma, primary squamous cell carcinoma (SCC) of the stomach is defined as a tumor consisting of SCC components and definitively generated from the stomach [1]. As the definition is very stringent, primary gastric SCC is rare, accounting for only 0.04–0.07% of all gastric cancers [2]. While there are some hypotheses about the origin of this tumor, its cell of origin is still unclear. Its rarity may be one of the reasons for this lack of knowledge. Moreover, primary gastric SCC is known to have a bad prognosis [3]. As the first step of improving prognosis, we have to accumulate more gastric SCC cases and analyze each case in detail.

We experienced a rare case of pure primary SCC of the stomach. In the course of diagnosis of this tumor, we performed histochemical and immunohistochemical studies. These studies showed interesting and suggestive results regarding the origin of these tumor cells. Herein, we report these results and try to examine the mechanism of tumor development in this case.

## Case presentation

**Fig. 1:**
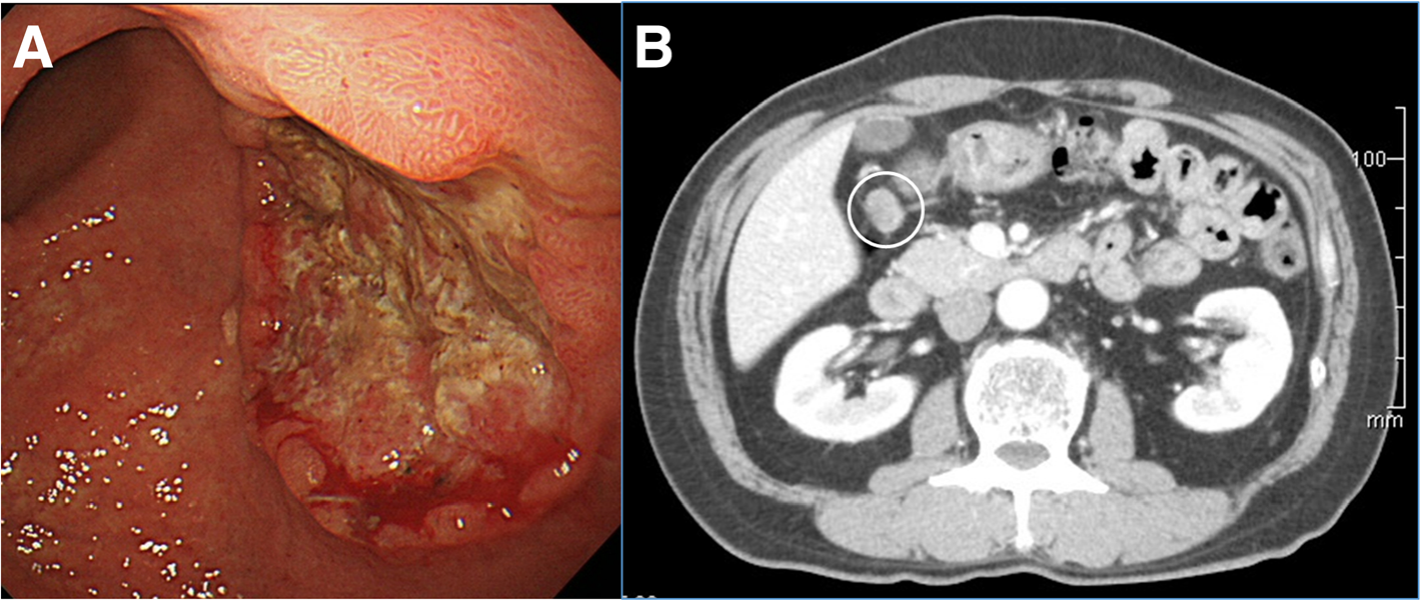
Esophagogastroduodenoscopy and computed tomography of the tumor. **a** Esophagogastroduodenoscopy revealed a type 2 lesion in the posterior wall of the lower body of the stomach. **b** Contrast-enhanced computed tomography indicated swelling of the perigastric lymph node (white circle) but showed no other distant metastasis

A 60-year-old man consulted a previous physician complaining of upper abdominal pain. Although he was prescribed H2 blocker, his symptoms did not improve, and esophagogastroduodenoscopy (EGD) was performed. EGD revealed a type 2 lesion in the posterior wall of the lower body of the stomach (Fig. 1a), and the biopsy indicated carcinoma. He was referred to our hospital for detailed examination.

On admission, abnormal symptoms, such as fever, anemia, and jaundice, were not observed, and his performance status was good (Eastern Cooperative Oncology Group score of 0). He had hypertension and hyperuricemia and was taking medication. He had a history of eradication of *Helicobacter Pylori* (HP). He had no history of smoking, and had no family histories of malignant diseases, either. Laboratory data on admission showed no remarkable findings, and tumor marker levels (carcinoembryonic antigen and carbohydrate antigen 19–9) were also not increased. The EGD and biopsy specimens were re-examined, and they revealed poorly differentiated adenocarcinoma. Contrast-enhanced computed tomography (CT) from the neck to the bottom of the pelvic floor indicated swelling of the perigastric lymph node (Fig. 1b) but showed no other distant metastasis.

Based on these findings, the patient was diagnosed with cT2N1M0, cStage IIA gastric adenocarcinoma (according to the Union for International Cancer Control (UICC) TNM classification of malignant tumors, 8th edition).

**Fig. 2:**
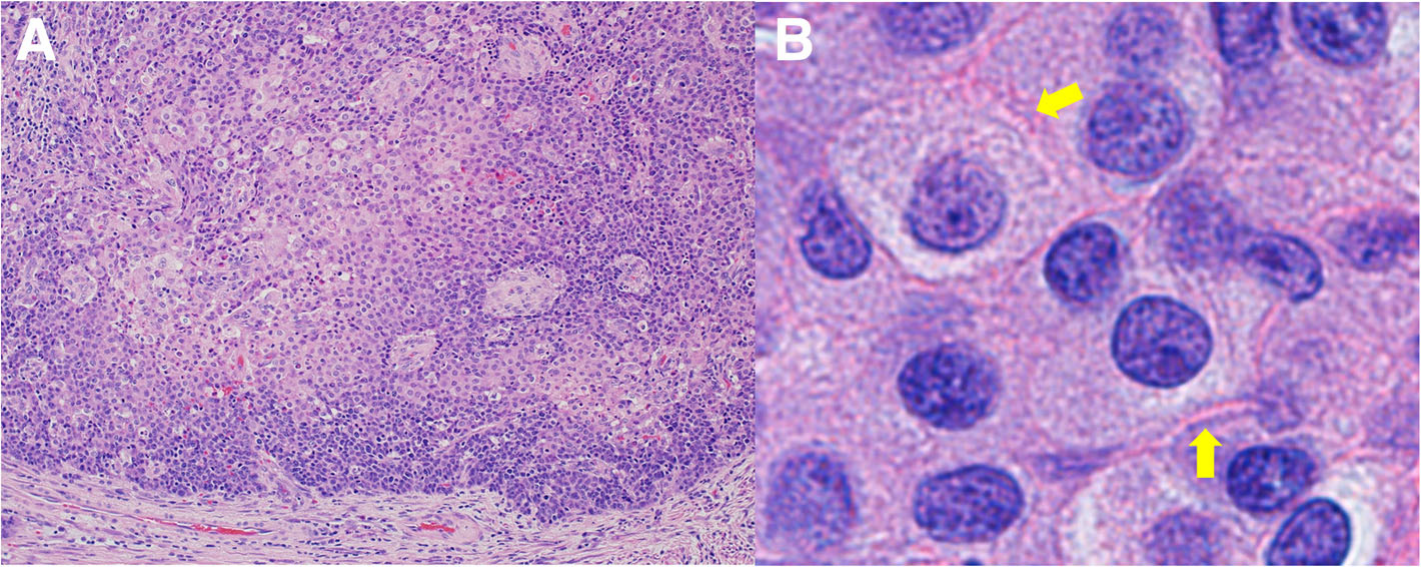
Hematoxylin and eosin staining of the resected tumor specimen. **a** Hematoxylin and eosin (HE) staining of the tumor specimen showed that the tumor cells had hyperchromatic nuclei and an abundant amount of eosinophilic cytoplasm, and proliferated in a sheet-like structure with solid nests (× 20). **b** We also detected intercellular bridges (yellow arrows) (× 100)

**Fig. 3:**
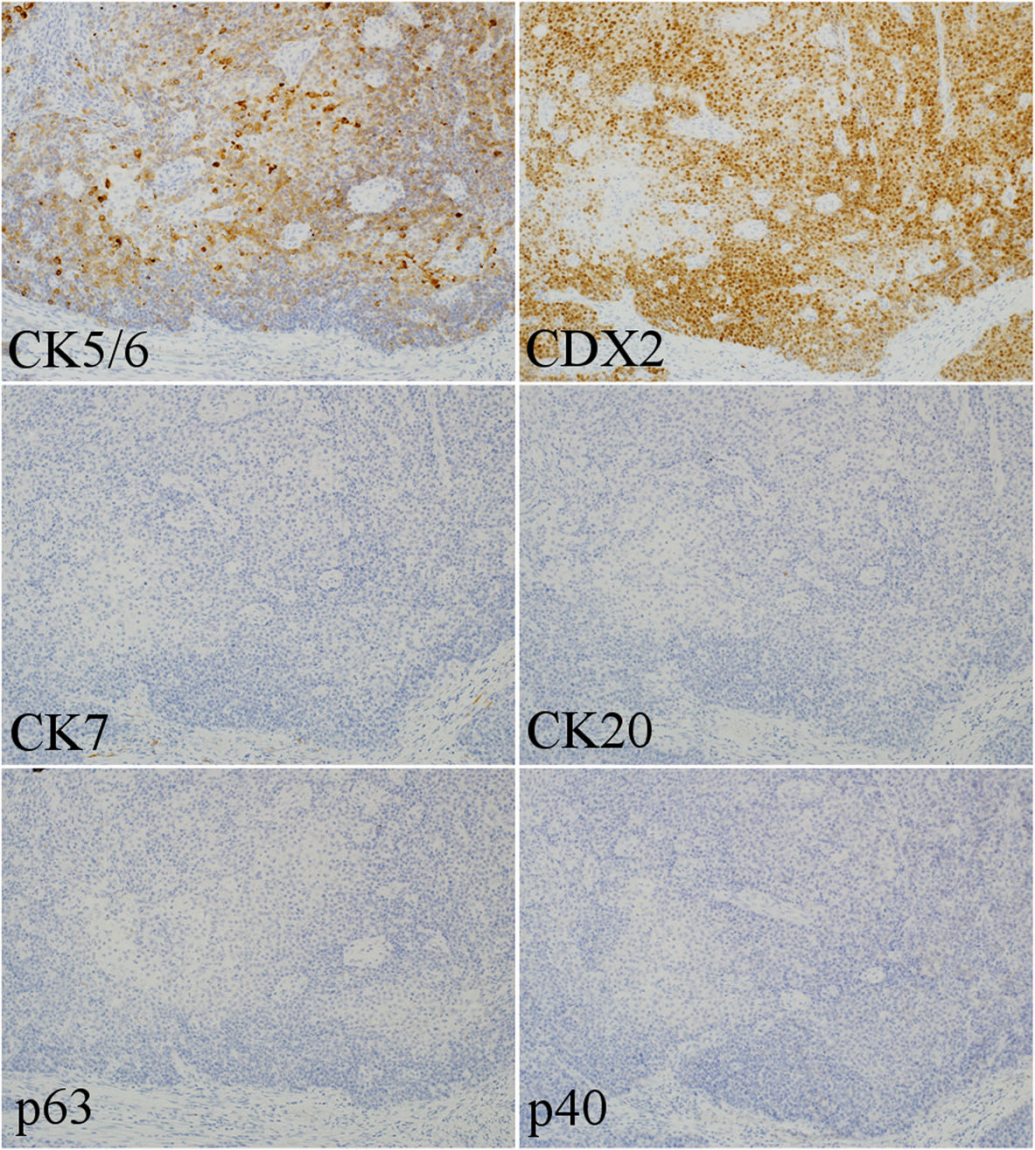
Immunohistochemistry of the tumor specimen (× 20). Immunohistochemistry of the resected tissue specimen was positive for cytokeratin (CK) 5/6, and caudal-type homeobox protein 2 (CDX2), slightly positive for CK20, and negative for p63, p40, and CK7

Distal gastrectomy with D2 lymph node dissection was performed. The primary lesion was 4 × 4 cm in size and extended into the subserosal layer. Thirty-eight lymph nodes were harvested, and there were 14 metastatic lymph nodes. Stations of metastatic lymph nodes were nos. 3a, 3b, 4d, 6, and 7. Although the preoperative biopsy suggested adenocarcinoma, histopathological examination of the resected specimen indicated SCC, and it was diagnosed as pT3N3aM0, pStage IIIB (according to the UICC TNM classification of malignant tumors, 8th edition). Hematoxylin and eosin (HE) staining of the tumor specimen showed that the tumor cells had hyperchromatic nuclei and an abundant amount of eosinophilic cytoplasm, and proliferated in a sheet-like structure with solid nests. We also detected intercellular bridges in some sections (Fig. 2). HE staining of the metastatic lymph nodes showed findings similar to those of the primary tumor. Atrophic change with intestinal metaplasia was observed in the mucous membrane around the tumor, and former infection of HP was suggested. And the tumor specimen did not show the pattern like lymphoid stroma, and infection of Epstein-Barr virus (EBV) was not suggested. Immunohistochemistry of the resected tissue specimen was positive for pan-cytokeratin (AE1/AE3), cytokeratin (CK) 5/6, and caudal-type homeobox protein 2 (CDX2), slightly positive for CK20, and negative for p63, p40, synaptophysin, α-fetoprotein, and CK7 (Fig. 3).

He was discharged without any complications 9 days after the operation. He received adjuvant chemotherapy with S-1 (100 mg/body/day of S-1 on days 1–28, every 6 weeks). After 5 cycles of S-1 therapy, a follow-up CT scan showed multiple metastases in the liver and around the duodenal stump. Then, he received two courses of CapeOX therapy (3000 mg/body/day of capecitabine on days 1–14 and 130 mg/m^2^ of oxaliplatin on day 1, every 3 weeks), but failed because of skin trouble. Furthermore, he received three courses of weekly paclitaxel and ramucirumab-combined therapy (80 mg/m^2^ of paclitaxel on days 1, 8, and 15 and 8 mg/kg of ramucirumab on days 1 and 15, every 4 weeks), but failed because of the tumor progression. At this time, CT showed worsening of the liver and duodenal stump metastases and the new lesion of multiple subcutaneous and distant lymph node metastases, and he noticed melena. We selected chemotherapy regimens according to that of the Japanese Gastric Cancer Treatment Guidelines but could not obtain desirable results. So we selected docetaxel, cisplatin, and 5-fluorouracil (DCF) therapy (60 mg/m^2^ of docetaxel on day 1, 60 mg/m^2^ of cisplatin on day 1, and 700 mg/m^2^ of 5-fluorouracil on days 1–5, every 4 weeks), according to the Japanese regimen of esophageal SCC. After two courses of DCF therapy, CT showed a significant reduction of metastatic lesions and his complaint of subcutaneous nodules and melena disappeared, and got efficacy evaluation of partial response (PR), according to the Response Evaluation Criteria in Solid Tumors (RECIST). After five courses of DCF therapy, his tumor was getting worse; hence, best supporting care (BSC) was selected. He died 1 year and 5 months after the operation.

## Discussion

Primary gastric SCC is rare, accounting for only 0.04–0.07% of all gastric cancers [2]. And primary gastric SCC is known to have a bad prognosis [3]. In this case, although curative resection was obtained and adjuvant chemotherapy was performed, the tumor recurred during adjuvant chemotherapy, and the prognosis was poor. After relapse, the tumor was treated by chemotherapy in accordance with the Japanese Gastric Cancer Treatment Guidelines, but it was ineffective. DCF, according to the chemotherapy regimen for esophageal cancer, that followed a previous chemotherapy showed a certain effect. This fact suggests that gastric SCC belongs to a different population that of gastric adenocarcinoma. And, for its rarity and bad prognosis, we have to accumulate more gastric SCC cases and analyze each case in detail as the first step of improving prognosis. Then, it is indispensable to pursue the mechanism of the disease in order to find out the fundamental treatment. And, as a starting point to consider the mechanism of the occurrence of this tumor, a histopathological approach may be helpful.

A histopathological approach is indispensable for the diagnosis of cancer. Boswell and Helwig determined that gastric SCC must satisfy one or more of the following histopathological diagnostic criteria: (a) keratinized cell masses with pearl formation; (b) mosaic pattern of cell arrangement, in which some cell borders are sharp, some show individual cell keratinization, and some are flattened, with elongated nuclei and intense cytoplasmic eosinophilia with a whorl pattern; (c) presence of intercellular bridges; or (d) a high concentration of sulfhydryl and/or disulfide groups, indicating the presence of keratin or prekeratin [2]. In our case, HE staining of the tumor specimen showed tumor cells with abundant eosinophilic cytoplasm, the formation of sheet-like structures and solid nests, and intercellular bridges; therefore, we diagnosed this tumor as SCC.

If the SCC is located in a place where squamous epithelium does not exist and SCC cannot arise naturally, we must determine whether the tumor is primary or metastatic. SCC occurring in the cardia or in conjunction with the esophagus is not considered to be primary gastric SCC; therefore, to be considered a primary lesion, the tumor must arise elsewhere in the stomach, and there should be no evidence of SCC in any other organs [4]. As mentioned in the introduction, the Japanese classification of gastric carcinoma defines primary gastric SCC as tumors consisting of SCC components definitively generated from the stomach [1]. In this case, preoperative CT revealed only the primary tumor and one enlarged perigastric lymph node, and we did not find any abnormality or metastatic lesion in the abdominal cavity during the operation. Therefore, we diagnosed this tumor as a primary SCC of the stomach.

Once the possibility of metastasis is excluded and it is confirmed that the tumor is primary gastric cancer, we should consider whether it is really SCC. As the histopathological examination of this biopsy specimen indicated adenocarcinoma, histopathology alone was insufficient to make a diagnosis of SCC. In such cases, an immunohistochemical approach is useful as supporting evidence. For the detection of SCC, immunostaining of CK5/6, p63, and p40 (an isoform of p63) should be performed [5, 6]. In contrast, for the detection of the origin of adenocarcinoma (including gastric cancer), we usually use a combination of CK7 and CK20 [5]. We also use CDX2 as a marker of intestinal adenocarcinoma [7]. The reactivity for these markers in gastric SCC is of course unknown. Hence, it will be inferred from the reactivity in esophageal carcinomas. In the previous report, esophageal SCC consistently expressed CK5/6 (in 98% of all cases) and p63 (100%) with strong reactivity, partially expressed CK7 (34%) and CDX2 (27%), but most of CK7 and CDX2 expression demonstrated weak reactivity [8]. In this case, the tumor was positive for CK5/6 and CDX2, slightly positive for CK20, and negative for CK7, p63, and p40. CK, expressed in the epithelium and epithelial-derived cells, is one of the proteins that form intermediate filaments. CKs function by forming heterodimers, and the combinations vary depending on the type of epithelial cell, so they are useful for differentiating epithelial cells and epithelial carcinoma cells. In this case, the results of immunostaining for CK5/6, CK7, and CK20 supported that this tumor was SCC.

A question remains: why, in this case, was the tumor negative for p63/p40 and positive for CDX2? Considering what cell types these markers are staining and where they are expressed helps us to infer the pathways of tumor development. There are several hypotheses for the development of primary gastric SCC [9], including (1) arising from the ectopic squamous epithelium [2, 10], (2) arising from the squamous metaplasia of the gastric mucosa [2, 11], (3) squamous differentiation from a pre-existing adenocarcinoma [12], and (4) arising from multipotent stem cells [9]. In the histopathological examination of this case, intestinal metaplasia was observed in the mucous membrane around the tumor, but there was no squamous metaplasia. Moreover, the entirety of the tumor consisted of SCC component, and adenocarcinoma component was not detected. Pathological report of the biopsy specimen revealed “adenocarcinoma,” but, with limited specimen, it was difficult to distinguish poorly differentiated carcinoma whether it is adenocarcinoma or SCC. In gastric cancer cases, Japanese pathologists usually diagnose these cases as poorly differentiated “adenocarcinoma,” because almost all of the gastric cancers are adenocarcinoma. We re-examined HE staining of the biopsy specimen, it showed poorly differentiated “carcinoma” and was akin to the part of the tumor specimen. From these findings, hypotheses (2) and (3) are not appropriate for this case. There is a well-known theory that, under the exposure of chronic inflammation, the transformation of tissue stem cells causes metaplasia and dysplasia of the epithelium, then epithelial cancer arises [13]. In this case, pathogens that could cause chronic inflammation might be HP, EBV, and HPV. As mentioned in the “Case presentation” section, the patient had a history of former HP infection and background gastric mucosa showed atrophic change with intestinal metaplasia. And chronic gastritis which is developed by HP is known to cause repetitive injury and repair of gastric mucosa, resulting in hyperproliferation and increase of mitotic error, then progress to gastric cancer [14]. From this point of view, hypothesis (4) seems not to be appropriate, but under the environment that is exposed to chronic inflammation or tissue damage due to infection; there may be a possibility that stem cells will directly become cancer skipping the step of metaplasia or dysplasia (hypothesis (4)). At this time, hypotheses (1) and (4) remain, and it is useful to next consider whether and where p63/p40 and CDX2 are expressed. *p63* is located on chromosome 3q27–29, and p63 acts as a switch for initiation of epithelial stratification in the embryonic epidermis [6]. p63 is consistently expressed in the nuclei of basal cells of normal epithelia, such as the skin, esophagus, tonsils, ureter, uterine cervix, vagina, prostate, breast, and bronchi [6]. The 3q27–29 region containing *p63* is amplified in many SCCs, and therefore p63 overexpression is observed in those SCCs [6]. The majority of the p63 amplified isoforms in SCC are the dominant-negative ΔNp63 forms called p40, and p40 staining is highly specific in those SCCs [6]. *CDX2* is a caudal-type homeobox gene, encoding a transcription factor that promotes proliferation and differentiation of intestinal epithelial cells [7]. CDX2 is present in the nuclei of epithelial cells of the intestine from the duodenum to the rectum, and the expression of *CDX2* mRNA is highly restricted to the intestinal epithelium [7]. CDX2 expression is also observed in intestinal metaplasia and gastric adenocarcinoma [15]. In this case, the tumor was negative for p63/p40 and positive for CDX2. As this suggests that the tumor cells were not derived from ectopic squamous epithelium but from intestinal metaplasia, hypothesis (1) can be excluded. In addition to these facts, the fact that tumor cells looked like homogeneous SCC suggested that these tumor cells might directly arise from stem cells in the intestinal metaplasia of the stomach.

## Conclusion

We experienced a rare case of primary gastric SCC. From the histochemical and immunohistochemical findings, we concluded that these tumor cells arose from stem cells in the intestinal metaplasia.
